# Resource Use and Costs of Nurse Navigator Support for Parents of High-Risk Infants After Discharge from a Neonatal Intensive Care Unit

**DOI:** 10.3390/children13050665

**Published:** 2026-05-09

**Authors:** Vercancy Wu, Myla E. Moretti, Kayla Esser, Natasha Henriques, Jennifer D. Zwicker, Julia Orkin, Eyal Cohen, Nathalie Major, Wendy J. Ungar

**Affiliations:** 1 Child Health Evaluative Sciences, The Hospital for Sick Children Research Institute, Toronto, ON M5G 0A4, Canada; vercancy.wu@sickkids.ca (V.W.); myla.moretti@sickkids.ca (M.E.M.); natasha.henriques@sickkids.ca (N.H.); julia.orkin@sickkids.ca (J.O.); eyal.cohen@sickkids.ca (E.C.); 2 Institute of Health Policy, Management and Evaluation, University of Toronto, Toronto, ON M5T 3M6, Canada; 3 The School of Public Policy, University of Calgary, Calgary, AB T2P 1H9, Canada; zwicker1@ucalgary.ca; 4 Edwin S.H. Leong Centre for Healthy Children, The Hospital for Sick Children Research Institute, Toronto, ON M5G 0A4, Canada; 5 Division of Paediatric Medicine, The Hospital for Sick Children, Toronto, ON M5G 1X8, Canada; 6 Complex Medical Care Program, Paediatric Medicine, Children’s Hospital of Eastern Ontario, Ottawa, ON K1H 8L1, Canada; nmajor@cheo.on.ca

**Keywords:** care coordination, NICU, resource use, costs, medical complexity

## Abstract

**Highlights:**

**What are the main findings?**

- NICU-admitted infants use a wide range of health and allied professional services in the first year post-discharge.
- A total of 13–23% of primary caregivers and 1–7% of their partners sought and paid for mental health care services to help them cope with their child’s condition.
- Care coordination and nurse navigator support may help families access the services their infant needs after transitioning to home.

**What are the implications of the main findings?**

- NICU-to-home care coordination must consider the mental health needs of parents.
- Public and private health plans must provide adequate coverage for equipment needed to care for and monitor the infant at home.

**Abstract:**

**Background:** Infants discharged home from a neonatal intensive care unit (NICU) often have multiple ongoing medical needs. The Coached, Coordinated, Enhanced Neonatal Transition (CCENT) program provides nurse navigator-led support for caregivers of high-risk infants through their first year after transitioning from the NICU to home. The objective was to compare health care resource use and costs between CCENT and standard care control groups post-discharge. **Methods:** Resource use and costs were collected at 4 months and 12 months post-discharge from families enrolled in the CCENT randomized controlled trial across Canada. Infant healthcare utilization and parent mental health service use and costs were analyzed from public health care system and family payer perspectives and were compared statistically between groups and within groups over time. **Results:** A total of 97 and 105 infants were randomized to the intervention and control groups, respectively. Significant reductions in use of medications and equipment were observed over time in both groups while use of allied health professionals decreased and emergency department (ED) visits increased for CCENT. Annual total healthcare costs per child to the public payer were $4135 (95% CI $2825, $5709) for the CCENT group and $4578 (95% CI $2246, $8356) for controls. The cost of delivering CCENT was $669 per family (SD $362). The average annual out-of-pocket cost per family was $724 (95% CI $467, $1024) for CCENT and $728 (95% CI $479, $1007) for controls. **Conclusions:** This study indicates the importance of considering patterns of healthcare utilization, program costs and costs to families when implementing NICU to home care interventions. Excluding the cost of a nurse navigator, costs to the healthcare system were not increased in the intervention group. Such a program may help families access appropriate care.

## 1. Introduction

Effective medical management in neonatal intensive care units (NICUs) and post-discharge contributes to improved survival of infants born preterm or with complex medical needs [1,2,3]. These infants face numerous medical and developmental challenges [2,4]. Post-discharge care traditionally involves scheduled appointments with primary and specialist care providers and, for high-risk infants, participation in a neonatal follow-up program focusing on neurodevelopmental assessment [5,6]. In addition to the high levels of psychosocial stress that parents experience while their infant is in the NICU [7,8,9], navigating the healthcare system and accessing needed services can be challenging and increase stress for parents in the post-discharge period [10,11,12]. Effectively addressing the needs of families requires a model of care that extends beyond the NICU to include the transition to home and the first year post-discharge [13,14,15].

The Coached, Coordinated, Enhanced Neonatal Transition (CCENT) program was developed to support parents throughout the NICU admission, transition to home, and the first-year post-discharge [16]. The CCENT intervention was integrated in the NICU and delivered by a trained nurse navigator, encompassing three core elements: coaching and psychosocial support for parents within an Acceptance and Commitment Therapy (ACT) framework [17,18], care coordination with the infant’s medical team, and education and guidance for managing typical medical and developmental challenges [13,19]. The bundled nature and flexibility of the CCENT intervention allowed the nurse navigator to tailor interactions to the unique needs of parents, while adhering to the intervention’s core components. In this way, CCENT may be superior to previously evaluated short-term interventions that focused only on mother–infant interactions or on family partnering and consultation [20,21]. Understanding the value and costs of a nurse navigator intervention to the health care system and to families is essential to inform funding and future adoption decisions. The study objectives were to compare healthcare resource use and costs between CCENT and standard care (control) groups for one year post-discharge from a public healthcare system and family payer perspectives.

## 2. Materials and Methods

### 2.1. Study Design

This cost analysis was conducted alongside the CCENT multi-centre pragmatic randomized controlled superiority trial carried out in seven level III NICUs in Ontario, Quebec, and British Columbia, Canada [16]. Participants at each site were randomized to receive CCENT or standard care. Randomization was conducted centrally and stratified by site. Healthcare resource utilization and out-of-pocket costs were collected at 4 months and 12 months following NICU discharge and analyzed from the public healthcare and family payer perspectives. The effectiveness of CCENT was assessed separately [16].

### 2.2. Comparators

In addition to standard care neonatal follow-up, caregivers enrolled in the CCENT intervention group received a structured, nurse-led intervention designed to support caregivers during the transition from the NICU to home. A single nurse navigator was employed at each of the seven NICU study sites. The CCENT program included three key components: (1) coaching and psychosocial support using an ACT framework, (2) care coordination to facilitate access to health care services, and (3) anticipatory education tailored to the needs of caregivers managing a medically complex infant [16]. The intervention was delivered through four in-person sessions while parents were admitted to the NICU, followed by a schedule of weekly, then monthly check-in phone calls where nurse navigators supported parents and answered questions after discharge [16]. Parents could engage with the nurse navigator beyond the scheduled calls.

Controls received standard post-discharge care which helps caregivers assess, monitor, and intervene early to identify and address potential developmental concerns post-discharge. It consisted of scheduled visits at 4–8 weeks, 4 months, 8 months, 12 months, 18 months, and 36 months, focusing on neurodevelopmental and medical assessments with referrals to additional services as needed.

### 2.3. Recruitment and Data Collection

Parents were eligible to participate in the study if their infants were classified as high-risk, defined as having conditions associated with prolonged NICU admission and high likelihood of future medical needs which could include risk factors that predict neurodevelopmental delay or impairment, medical complexity, and impairment in parent–infant attachment. Detailed eligibility criteria can be found in the published protocol [16]. Eligible infants were those admitted to a participating level III NICU. Eligible caregivers were approached by study personnel prior to NICU discharge.

Data were collected between July 2019 and December 2022 at enrollment (baseline), 4 months post-discharge and 12 months post-discharge. Primary caregivers reported their ethnic identity, as well as sex and gender. Demographic and resource use questionnaires were self-completed by parents either online using a centralized Research Electronic Data Capture (REDCap) database [22] or by telephone interview with a research assistant. In addition, the nurse navigator recorded the frequency, duration and purpose of every interaction between the participant and the nurse navigator at each site. Additional details regarding data management are provided in Appendix A.

### 2.4. Resource Use and Costs

Post-discharge healthcare resource use and out-of-pocket costs were collected prospectively using a structured resource use questionnaire (RUQ) adapted by the study team from a tool validated for use in children with developmental conditions [23]. The RUQ captured detailed information on infant healthcare service utilization, including primary care visits, medical sub-specialist consultations, allied health services (complementary medicine therapists and nurses), emergency department (ED) visits, hospital readmissions, outpatient medications, medical materials and equipment (e.g., feeding pumps, oxygen tanks and monitors), and community-based care coordination services (external to CCENT). Out-of-pocket expenses related to each of these categories were also recorded. In addition, the RUQ captured primary caregiver and partner mental health service use and associated out-of-pocket costs, as well as time lost from paid or unpaid labour and usual activities related to the infant’s medical care needs. Detailed descriptions of methods used for pricing and costing are provided in Appendix A and in the Methods Supplement.

The CCENT intervention was delivered by dedicated nurse navigators. The cost of this intervention included fixed and variable components. Upfront fixed costs included expenses related to ACT training workshops, program development and delivery, and online meeting software licences and equipment (e.g., phones and computers). A fixed cost per family was calculated by dividing the total fixed cost by the planned enrollment target of 200 families. Variable costs were calculated at the family level reflecting interactions between each participant and their assigned nurse navigator. Interactions were flexible and based on parent needs. Total interaction time per family was multiplied by the nurse navigator’s hourly wage (mid-level, as reported by the Ontario Nurses’ Association) [24] to estimate the total variable cost per family. Costs were reported in 2025 Canadian dollars (CAD) (1 USD = 1.4 CAD).

### 2.5. Handling of Missing Data

The proportion of missing RUQ data ranged from 0% to 8%, depending on the variable. Missingness was attributed to non-response, lack of service utilization, incomplete cost reporting, and missing RUQ completion dates. Decision rules were applied to impute missing values where appropriate. Blank cost fields were recoded as zero if no service use was indicated. For cases where service frequency was reported but the service type was missing, either an average cost across similar services (e.g., all allied health professionals) or a conservative estimate was applied (e.g., assigning the primary care provider rate for an unspecified medical sub-specialist). When RUQ dates were missing, dates from other study instruments administered at the same assessment were used. Imputation of missing nurse navigator interaction time was conducted using the grand mean of total interaction time per family from all sites.

### 2.6. Measurement and Valuation

Parents reported the frequency and duration of each type of resource used since discharge at the 4-month assessment and since the 4-month assessment at 12 months post-discharge. Within each recall period, the use of each service was converted to a monthly frequency and multiplied by the number of months the service or resource was used. All resource use was assigned to the child as the unit of analysis. In the case of families with twins, parental time losses, parental mental service use and care coordination service use reported for one or both twins were allocated across both twins. Costs for each service category were determined by multiplying unit prices by volume of use per child for each recall period. A total cost per child was calculated for participants in each group by summing costs across all resource categories. Mean costs per child were calculated for each group from public payer and family payer perspectives. Due to the right-skewed distribution of cost data and the presence of zero values, 5000 bootstrapped samples were generated by resampling data with replacement within each study group using Microsoft Excel Visual Basic for Applications (version 2021). For each bootstrapped sample, mean total costs per child for the intervention and control groups, as well as the between-group incremental total costs, were calculated. Ninety-five percent confidence intervals (CI) were obtained using the 2.5th and 97.5th percentiles of the bootstrapped distributions for all means. CCENT intervention costs were determined with the family as the unit of analysis and were calculated by summing fixed and variable costs per family. Detailed costing methods for each type of service are provided in Appendix A.

Since participants reported resource use over variable durations since discharge, all costs were standardized to an annual estimate of costs per child during the first-year post-discharge by determining the monthly cost over the reporting period and multiplying by 12. Only cases that reported resource use at both assessments were included in the annualized cost analysis.

The public payer perspective included infant medical services such as primary care and sub-specialist services, emergency department visits, medications and publicly funded allied health professional services, such as prescribed homecare nurses. Parent mental health services funded by provincial healthcare programs, and costs related to provincial care coordination services, such as social workers, were also included in the public payer perspective.

The family payer perspective included out-of-pocket expenses incurred by the family for their infant’s medications, ambulance services, equipment and materials directly contributing to the infant’s care, allied health professional services, private care coordination, and parental mental health service costs that were not reimbursed.

A conservative approach was taken whereby care coordination services that were reported by parents as “care coordinator” or “navigator” were excluded from the reference case analysis for both groups due to possible overlap with the intervention which was costed separately. Travel costs were excluded due to variable reporting and the potential for recall error. Infant readmission costs were examined in an exploratory analysis. This high-risk patient population experienced frequent readmissions with wide variability in intensity and duration of care. This contributed to extremely high case costs. The primary reason for admission was based on parent report. Readmission costs were ultimately excluded from the calculation of total annual costs due to uncertainty in estimating accurate costs from parent-reported reasons for admission and the high variability (both intensity and duration) of such admissions, which could introduce substantial measurement error into total cost estimates thereby hampering the ability to detect any effect of CCENT on ambulatory care services.

### 2.7. Statistical Analysis

Descriptive statistics were used to summarize participant demographic and medical characteristics and resource use. A descriptive analysis of nurse navigator interactions was conducted for participants in the intervention group. This included the distribution of the total number of interactions per family, the average time spent per interaction, the purpose of each interaction, the care coordination need addressed, and the type of action taken. Differences in individual categories of resource use and the annual cost per child between CCENT and standard care groups were assessed using two-tailed Student t-tests for continuous variables and chi-square tests or Fisher’s exact test for categorical variables. The significance of differences in mean total costs between groups from bootstrapped iterations was assessed by examining 95% CIs for incremental values.

### 2.8. Sensitivity Analysis

One-way deterministic sensitivity analyses were undertaken to evaluate the effects of uncertainty in select unit prices and intervention-specific components. First, wages for allied health professionals were varied within plausible ranges. Second, parental mental health service costs were varied independently to account for differences in provider types and associated billing rates. Third, care coordinator wages were varied to reflect differences in qualifications and employment models. Fourth, alternative combinations of care coordinator professionals within care teams were tested.

For the intervention group, three additional independent one-way sensitivity analyses were performed. These included varying the nurse navigator’s hourly wage, total time spent delivering services per family, and the allocation method of the nurse navigator’s fixed startup cost. Additional details are provided in Appendix A.

## 3. Results

### 3.1. Sample Demographics

**Figure 1:**
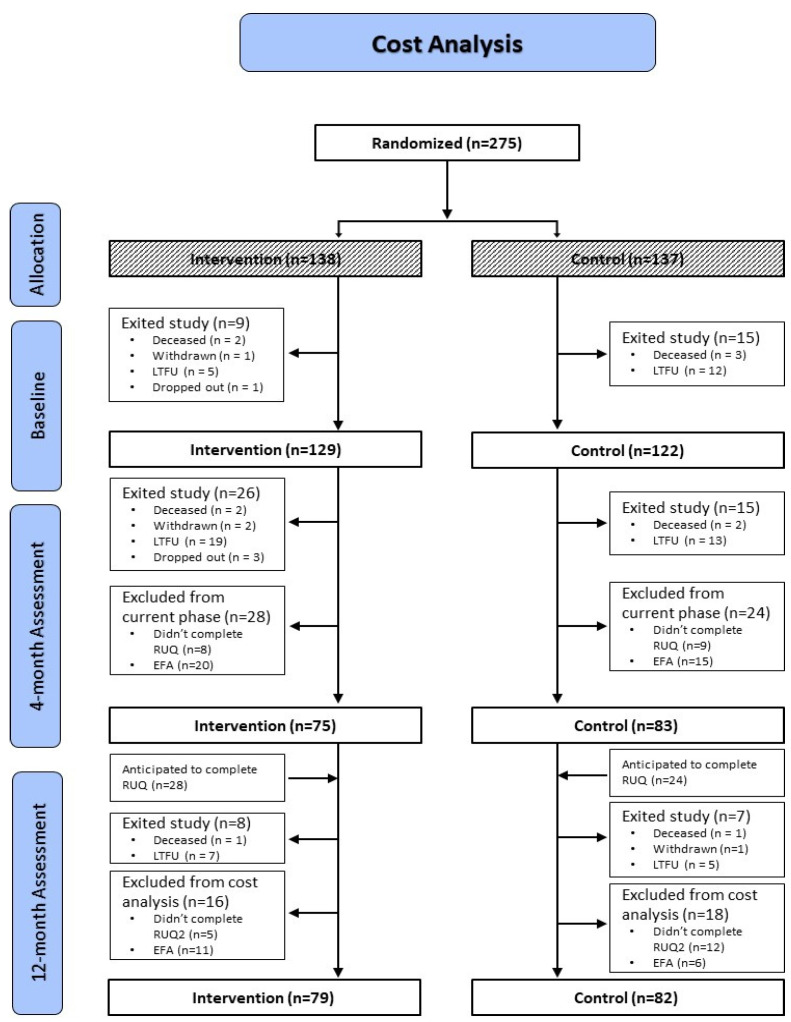
Cost analysis CONSORT chart. Abbreviations and definitions: Lost to follow up (LTFU): Unable to contact participant after 3 calls and 3 e-mails; Dropped out: Parent asked to be removed from study; Withdrawn: Removed by study team due to ineligibility criteria post-enrollment; EFA: Excluded from analysis due to incomplete/invalid data or failure to complete RUQ within required interval.

Of 1028 families meeting eligibility criteria, 504 families (49%) were invited to participate (Supplemental Figure S1). Of those invited, 275 families were randomized: 138 to the intervention arm and 137 to the control arm. Baseline demographic data were available from 94 parents of 97 children in the intervention group and 101 parents of 105 children in the control group. Of these families, cost data were available from parents of 75 (54%) intervention arm infants and from 83 (61%) control arm infants at 4 months post-discharge, and from parents of 79 (57%) intervention arm infants and 82 (60%) control arm infants at 12 months post-discharge (Figure 1).

Baseline child and parent characteristics are summarized in Table 1. Most infants were born at less than 32 weeks of gestational age (GA) and had six or more co-diagnoses. Most parents reported living in a single child household. Approximately half of parent participants were born outside of Canada and most reported non-European ancestry.

### 3.2. Resource Use

High rates of resource use for primary and specialty care as well as allied health professionals were reported in both groups at 4 months and 12 months post-discharge (Table 2). Use of allied health professional services were significantly higher in CCENT compared to controls at 4 months, primarily due to greater use of homecare nurses and occupational therapists (*p* < 0.05) (Supplemental Table S5.2). In the CCENT group, use of allied health professionals decreased from 52% to 37% (*p* < 0.05), prescription medicines from 49% to 33% (*p* < 0.05) and purchased equipment and materials from 53% to 28% (*p* < 0.005) over time. ED visits increased from 25% to 41% over time in the CCENT group (*p* < 0.05). Infant admission likewise increased in the CCENT group but the difference was not statistically significant. The control group exhibited statistically significant decreases in purchased equipment and materials and medications over time (*p* < 0.05). While approximately half of all parents in both groups reported a productivity time loss to care for their infant, it was expected that unreported parental leave would also have been devoted to infant caregiving. Tables summarizing each category of resource use are available in Supplemental Tables S1–S10.

### 3.3. Costs

Costs to the public payer for common categories of resource use were right-skewed due to a small proportion of participants reporting high costs. As seen in Table 3, the annual cost per child was highest for allied health professionals and parental mental health service use. The mean total annual cost per child to the public payer was $4135 (95% CI $2825, $5709) for the intervention group and $4578 (95% CI $2246, $8356) for the control group. The intervention group was on average less costly due to lower use of allied health professionals but the difference −$442 (95% CI −$4448, $2568) was not statistically significant.

Costs incurred by the families for common categories of resource use were similarly right-skewed. As seen in Table 4, the annual cost per child was highest for purchased equipment and materials and parental mental health service use. The mean total annual cost per child to the family was $724 (95% CI $467, $1024) for the intervention group and $728 (95% CI $479, $1007) for the control group. The difference between groups, −5 (95% CI −383, 382), was not significant.

**Figure 2:**
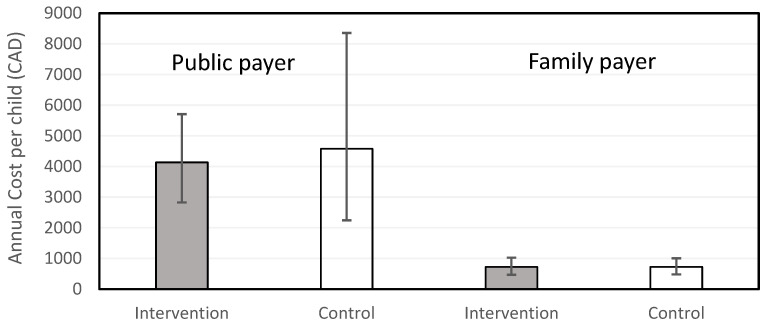
Mean total cost per child by group and payer perspective. Error bars represent 95% confidence intervals derived from bootstrapped estimates.

The mean total costs per child for each group from the public and family paper perspectives are displayed in Figure 2.

Analysis of nurse navigator use in the intervention group revealed a mean of five interactions per family per month (SD 4.1) over the first 4 months post-discharge (Supplemental Table S11.0). Consistent with the protocol, this rate tapered to 2.2 per family per month (SD 2.6) over the ensuing 8 months. For 90% of families, interactions with nurse navigators were characterized as having a mental health and well-being objective (Supplemental Table S11.1). The mean cost of a nurse navigator per family was $669 (SD 362) and ranged from $275 to $2429 (Supplementary Table S11.5).

### 3.4. Sensitivity Analysis

Table 5 presents the impact of varying uncertain unit prices on the incremental costs per child and on the intervention cost per family. When a minimum hourly wage was used for allied health professionals, including complementary medicine therapists and nurses, savings for the intervention group were reduced by 69%. Using maximum hourly wages resulted in a 58% increase in savings between the intervention and control group mean cost per child. This was due to higher volumes of allied health professional use in the control group, thus as wages decreased, the savings disproportionately benefited the control group. In contrast, when minimum hourly wages of mental health practitioners were used to cost parental mental health visits to psychologists, psychotherapists and family counsellors, incremental savings increased by 16% due to greater use of these services in the intervention group. When reported care coordinator and patient navigator use was included in the costs for both groups, incremental savings were reduced by 7%. Incremental costs were insensitive to variations in the hourly wage of care coordinators and patient navigators. The cost of delivering CCENT per family was very sensitive to the number of hours individual nurse navigators spent with a family and the number of families served per nurse navigator.

## 4. Discussion

An integrated care approach that includes a nurse navigator may help families transitioning from the NICU to home by providing coaching, psychosocial support, guidance and education, and by facilitating time access to appropriate services during the first year post-discharge. The infants in this sample were high-risk, with many requiring follow-up with multiple specialists. In this context, nurse navigator-led care coordination may increase use of appropriate services and therefore may not reduce short-term costs. Resource use patterns changed over time in both the CCENT and control groups. In the first four months post-discharge, infants in the CCENT group demonstrated significantly greater use of allied health professional services such as home care nurses compared to controls. The difference between groups attenuated over time. Both CCENT and control infants’ use of prescription medications and equipment and materials decreased significantly over time. Rates of ED visits unexpectedly increased over time for the CCENT group. The increased number of ED visits in both study groups, and especially the intervention group, was due to higher rates of fever/infection and respiratory problems at 12 months. In addition, some infants in the intervention group experienced more ED visits for device problems or medical procedures. One possible explanation is that more CCENT parents took their infants to the ED after consulting their nurse navigator and some of these cases may have been admitted for observation. An important function of nurse navigators is to provide guidance to parents seeking care for their child.

Total annual public payer costs per child were highly variable, but mean costs were similar between groups. The results showed costs of over $4000 per year in addition to any inpatient care costs which were excluded from the analysis. If the public payer were to fund CCENT, an additional cost exceeding $700 per family would be incurred. In reality, nurse navigators work part-time in this capacity while allocating their paid hours across a range of NICU and neonatal follow-up activities, which would reduce intervention costs.

Despite most costs falling on the public payer in a publicly-financed healthcare system, parents incurred mean out-of-pocket costs exceeding $700 per year that were not reimbursed by public or private health insurance. Most of these expenditures were for private mental health services for parents and for outlays in the home to accommodate their child’s needs.

Very few studies have investigated resource use and costs for care coordination interventions post-discharge from NICUs. In a randomized controlled trial of comprehensive post-discharge follow-up care for very-low birth weight infants, Broyles et al. found that surviving infants who received comprehensive post-discharge care demonstrated significantly more clinic visits and more clinic staff interactions than controls [25]. Outpatient costs in the year post-discharge were USD 2748 (SD 2415) per child in the intervention group compared to USD 2931 (SD 3635) in controls, but that study only costed follow-up clinic visits and not other types of specialist services or services used by parents. In that study, overall savings were observed due to reduced inpatient visits in the intervention group [25]. A more recent study examined resource use and costs associated with receipt of enhanced support services delivered by social workers and family resource specialists before and after NICU discharge [26]. Infants in the intervention group demonstrated significantly lower Medicaid spending compared to controls due to fewer ED visits and readmissions. Like the Broyles study, that study did not measure ambulatory care services.

In a study examining health service use before and after receipt of care coordination in children with medical complexity, Peter et al. observed significant reductions in ED visits, hospital admissions and costs, but that study did not include a control group comparator [27]. In a study of care coordination for persons with chronic disease aged 0–25 years, healthcare service use and annual Medicaid expenditures showed similar non-significant reductions for both study groups [28]. Finally, in a randomized trial comparing care coordination delivered by a nurse practitioner–pediatrician dyad to families of children with medical complexity (median age 24 months), participants receiving care coordination were slightly more likely to report use of home care (95% vs. 84%, *p* < 0.05) [29]. In addition, a median of >10 visits to specialists in the first year were observed for both groups. First year healthcare costs were similar between groups, CAD 52,804 for the intervention group compared to CAD 50,176 for controls [29]. The present analysis that demonstrated high rates of heterogenous service use in high-risk infants, with higher rates for allied health professionals such as home care nurses in the intervention group, are consistent with these published findings.

Previous studies largely focused on infant healthcare service use and costs and did not measure parental use of mental health services or family-incurred costs. Parents of NICU infants experience high stress levels [9], and while interventions such as CCENT that incorporate the principles of ACT can improve parents’ abilities to cope with difficult events [18], many parents will also seek professional mental health services. In addition to infant healthcare use, the present study captured data related to parent mental health services and found that 19–23% of primary caregivers and 5–7% of secondary caregivers sought professional mental health services during the 4 months post-discharge. Despite lower utilization rates compared to other categories of resource use, costs to the public payer for mental health services were among the highest and most variable, reaching a maximum of approximately $20,000 per couple per year for both groups. Parents’ average annual out-of-pocket costs for private mental health services exceeded $100 per couple, with some parents incurring costs exceeding $2500. Parents of infants in the NICU have been found to experience high stress levels [7,8,9] as well as anxiety and depression [30,31,32] and these effects may heighten during the transition to home [10,11]. An approach that considers not only the infant’s acute and long-term needs, but also psychosocial support for parents is essential in a family-centred model of care within publicly funded healthcare systems [13,14].

This study had several strengths and limitations. While this was a randomized design with high internal validity, the potential for selection bias whereby consenting families had different levels of resource use and costs than those who were eligible but declined would reduce external validity. In addition, high levels of clinical heterogeneity may have resulted in between-group differences in medical characteristics associated with post-discharge use of health services, particularly ED visits and hospitalizations. The sample size precluded sub-group analysis to explore these impacts further. The prospective design enabled the collection of detailed health service use data as well as data regarding use of allied health professionals, outlays for purchased equipment and parental time losses—data that cannot be obtained from other sources such as claims or administrative databases. While these data help contribute to original findings, the stress that families with an infant in the NICU experience can make it difficult to recruit participants and maintain their involvement. Many parents only completed either a 4-month or a 12-month assessment or did not complete the assessments within required intervals. This resulted in fewer cases available for the full-year cost analysis. A total of 22% of participants who provided baseline demographics data did not complete the 4- or 12-month RUQs. Reasons for attrition in both study groups included infant death, withdrawal from the study and lost to follow-up (Figure 1). Some cases were excluded from the cost analysis due to RUQ completion not falling within required intervals. Reasons for attrition were similar between groups. While this reduced statistical power, the sample size remained adequate to detect differences between groups in rates of resource use. The significance of differences in costs was determined by examination of 95% CIs from bootstrapped replicates. Group sample sizes remained stable between the 4- and 12-month assessments. The use of imputation and decision rules for missing data in continuing participants contributed to the robustness of the data available for analysis. While families receiving CCENT demonstrated greater use of allied health professionals such as home care nurses compared to controls at 4 months, controls received usual care coordination. This may have dampened the ability to detect effects of the CCENT nurse navigator on other types of service use. The disaggregated and transparent presentations of sample characteristics, resource use for each type of service, and item prices, facilitate comparisons to other high-risk NICU populations in other jurisdictions. This in turn allows policy and funding decision-makers to judge to what extent the findings may be applicable to their regions. Generalizability may be limited to jurisdictions with similar healthcare systems, NICU services and family demographics.

## 5. Conclusions

Infants in this study used a wide range of health and allied professional services in the first year post-discharge and some parents sought and paid for mental health care services for themselves. NICU integrated care coordination and nurse navigator support did not result in increased costs to the public payer (excluding the cost of a nurse navigator) and may help families access the services their infants need after transitioning to home. These programs must consider the mental health needs of parents. Public and private health plans must also provide adequate coverage for equipment needed to care for and monitor infants at home.

## Figures and Tables

**Table 1:** Sample characteristics at baseline.

Child Characteristic	Intervention (n = 97) ^1^		Control (n = 105) ^2^	
	Count	%	Count	%
Prematurity				
Birth ≤ 26 weeks of GA	23	24%	18	17%
Birth between 27 and 32 weeks of GA	42	43%	48	46%
Birth > 32 weeks of GA	32	33%	39	37%
Mean GA, weeks (SD)	27.91 (4.92)		28.95 (4.78)	
Medical complexity				
Medical complexity ^3^	14	14%	20	19%
>2 congenital anomalies ^4^	17	18%	16	15%
Other	6	6%	12	11%
None	60	62%	57	54%
Mean number of clinical diagnoses (SD)	7.47 (3.72)		6.84 (3.75)	
Number of unique clinical diagnoses				
0	1	1%	0	0%
1–5	30	31%	43	41%
6–10	49	51%	48	46%
11–15	13	13%	11	10%
>15	4	4%	3	3%
Female sex at birth	53	55%	60	57%
Achieved median percentile				
Neonatal length		32.1%		34.0%
Neonatal weight		43.6%		46.4%
Neonatal head circumference		37.9%		38.7%
**Parent Characteristic**	**Intervention (n = 94)**		**Control (n = 101)**	
	**Count**	**%**	**Count**	**%**
Mother (biological)	90	96%	97	96%
Mean age of mother, years (SD)	32.30 (5.65)		32.27 (5.51)	
Marital status				
Married or common law	77	82%	68	67%
Never married	11	12%	9	9%
Other (divorced/not specified/ prefer not to say)	6	6%	8	8%
Number of children in household				
1	75	80%	75	74%
2	10	11%	20	20%
>2	9	10%	6	6%
Language most often spoken at home				
English	70	74%	62	61%
Other ^5^	46	49%	48	48%
Immigrant status				
Born in Canada	49	52%	41	41%
Immigrated < 3 years earlier	15	16%	16	16%
Immigrated 3–10 years earlier	14	15%	19	19%
Immigrated > 10 years earlier	16	17%	25	25%
Ancestry				
First Nations, Inuit or Metis	3	3%	4	4%
Any European	27	29%	34	34%
North and other African	10	11%	8	8%
Caribbean	9	10%	5	5%
South Asian, Central Asian or Middle eastern	17	18%	16	16%
East and Southeast Asian	12	13%	19	19%
Other ^6^	16	17%	15	15%
Education				
High school or less	11	12%	10	10%
College undergraduate or less	61	65%	53	52%
Graduate degree	16	17%	23	23%
Other/prefer not to say	6	6%	15	15%
Employment status				
Working full time	17	18%	16	16%
Working part time	3	3%	1	1%
Laid off, sick or parental leave	57	61%	59	58%
Other ^7^	17	18%	25	25%
Annual household income				
Under $50,000	23	24%	26	26%
$50,000 to $99,999	32	34%	31	31%
>$100,000	31	33%	32	32%
Not specified/prefer not to say	8	9%	12	12%

Abbreviations: GA, gestational age; SD, standard deviation. ^1^ Includes 3 pairs of twins (6 infants), data for individual infants entered separately. ^2^ Includes 4 pairs of twins (8 infants), data for individual infants entered separately. ^3^ Infants born between 27 and 29 + 6 weeks GA who required invasive or non-invasive procedures at 34 weeks GA and/or supplemental oxygen at 37 weeks GA. ^4^ As defined by EUROCAT (version 1.3) (e.g., congenital heart disease, spina bifida, cleft palate) not including minor congenital anomalies (e.g., dysmorphic facies) and a minimum length of stay of 14 days in the recruiting institution. ^5^ French, Arabic, Bengali, Chinese, Cree, Filipino, German, Gujarati, Hindi, Hungarian, Italian, Korean, Mandarin/Cantonese, Persian, Polish, Portuguese, Punjabi, Russian, Somali, Spanish, Tagalog, Tamil, Ukrainian, Urdu, Vietnamese, Bafang, Hebrew, Yoruba, ASL—American Sign Language, Dutch, Punjabi, Edo, Malayalam, not specified/prefer not to say. ^6^ Oceania origins (Australia, New Zealand, Fiji), Caucasian, Black, Asian, White, Canadian, Caucasian, Latin Ventral and South American origins, Japanese Canadian, not specified/prefer not to say/unknown. ^7^ Student, homemaker, looking for work, unemployed, receiving disability, not specified/prefer not to say.

**Table 2:** Overall resource use by group and recall period.

Category	4 Months (T1)				12 Months (T2)			
	Intervention (n = 75)		Control (n = 83)		Intervention (n = 79)		Control (n = 82)	
Primary health care	68	91%	79	95%	70	89%	74	90%
Medical sub-specialist	42	56%	43	52%	46	58%	51	62%
Emergency department ^a^	19	25%	21	25%	32	41%	27	33%
Inpatient admission	14	19%	14	17%	23	29%	17	21%
Allied health professional *^,a,d^	39	52%	31	37%	29	37%	28	34%
Prescription medications ^a,c^	37	49%	48	58%	26	33%	34	41%
Purchased equipment and materials ^b,c^	40	53%	42	51%	22	28%	27	33%
Time loss by primary or secondary caregiver	37	49%	47	57%	38	48%	44	54%
Mental health services (survey respondent) **	17	23%	9	11%	15	19%	11	13%
Mental health services (spouse/partner) **	5	7%	1	1%	4	5%	1	1%
Care coordination service	8	11%	4	5%	8	10%	4	5%

* Allied health professionals included complementary medicine therapists and nurses. ** Mental health service providers included psychiatrists, psychologists, psychotherapists, marriage and family therapists, primary mental health care providers and group therapy. ^a^ Intervention group T2 vs. T1, *p* < 0.05. ^b^ Intervention group T2 vs. T1, *p* < 0.005. ^c^ Control group T2 vs. T1, *p* < 0.05. ^d^ T1 intervention vs. control, *p* < 0.05.

**Table 3:** Annual cost per child by group from the public payer perspective.

Category	Intervention (n = 57)					Control (n = 60)				
	Mean Cost per Child	SD	Median	Min	Max	Mean Cost per Child	SD	Median	Min	Max
Primary health care	595	419	472	165	2247	548	256	519	133	1357
Medical sub-specialist	303	376	182	0	1570	351	417	225	0	1864
Emergency department	554	1151	0	0	6843	413	690	0	0	3306
Allied health professional *	1027	1873	358	0	10,690	2136	11,371	0	0	87,891
Prescription medications	123	213	21	0	843	78	157	27	0	882
Purchased equipment and materials	0	0	0	0	0	0	0	0	0	0
Mental health services for both parents **	1505	3945	0	0	21,782	992	3250	0	0	19,543
Care coordination service	37	140	0	0	688	55	265	0	0	1605
Mean total cost	4144	5549	2000	218	24,818	4573	12,983	1423	137	98,012
Bootstrapped mean total cost (95% CI)	4135 (2825, 5709)					4578 (2246, 8356)				
Bootstrapped incremental costs (Intervention–Control) (95% CI)	442 (−4448, 2568)									

Abbreviations: SD, standard deviation; Min, minimum; Max, maximum; CI, confidence interval. All costs in 2025 CAD. * Allied health professionals included complementary medicine therapists and nurses. ** Mental health service providers included psychiatrists, psychologists, psychotherapists, marriage and family therapists, primary mental health care providers and group therapy.

**Table 4:** Annual cost per child by group from the family payer perspective.

Category	Intervention (n = 57)					Control (n = 60)				
	Mean Cost per Child	SD	Median	Min	Max	Mean Cost per Child	SD	Median	Min	Max
Primary health care	0	0	0	$0	$0	$0	$0	$0	$0	$0
Medical sub-specialist	0	0	0	$0	$0	$0	$0	$0	$0	$0
Emergency department	10	33	0	$0	148	9	40	0	0	259
Allied health professional *	82	413	0	$0	2894	41	199	0	0	1381
Prescription medications	66	145	0	$0	703	60	130	0	0	808
Purchased equipment and materials	410	757	33	$0	3784	464	859	69	0	4156
Mental health services for both parents **	154	624	0	$0	3953	126	510	0	0	2699
Care coordination service	0	0	0	$0	0	28	220	0	0	1702
Total cost	722	1097	167	$0	4360	728	1083	191	0	4156
Bootstrapped mean total cost (95% CI)	724 (467, 1024)					728 (479, 1007)				
Bootstrapped incremental costs (Intervention–Control) (95% CI)	−5 (−383, 382)									

Abbreviations: SD, standard deviation; Min, minimum; Max, maximum; CI, confidence interval. All costs in 2025 CAD. * Allied health professionals included complementary medicine therapists and nurses. ** Mental health service providers included psychiatrists, psychologists, psychotherapists, marriage and family therapists, primary mental health care providers and group therapy.

**Table 5:** Sensitivity analysis results from the public payer perspective.

Variable	Min	Max
Incremental cost per child, Intervention–Control		
Reference case	−429.23	
Allied health professional wage	−133.64	−679.37
Mental health practitioner wage	−497.70	−368.89
Addition of PN and CC in care coordination service	−399.62	
Intervention cost per family		
Reference case	865.21	
Nurse navigator hourly wage ($37.20 to $53.28)	802.67	948.15
Nurse navigator interaction hours per family (1 to 20)	510.01	1348.15
Number of families served per NN (42 to 138)	1708.26	797.69

Abbreviations: CC, care coordinator; NN, nurse navigator; PN, patient navigator. All costs in 2025 CAD.

## Data Availability

The original contributions presented in this study are included in the article/Supplementary Material. Further inquiries can be directed to the corresponding author.

## Supplementary Materials

The following supporting information can be downloaded at: https://www.mdpi.com/article/10.3390/children13050665/s1. Methods Supplement: Supplementary Costing Methods_wu3.docx; Supplemental Figure S1: Supplemental Figure S1_wu1.docx; Supplemental Figure S2: Supplemental Figure S2_wu8.docx; Supplemental Tables S1–S12: CCENT study Supplemental Tables 0-12_wu17.xls.

## Abbreviations

The following abbreviations are used in this manuscript:

ACT	Acceptance and Commitment Therapy
CAD	Canadian Dollars
CC	Care Coordinator
CCENT	Coached, Coordinated, Enhanced Neonatal Transition
CI	Confidence Interval
DNA	Deoxyribonucleic Acid
ED	Emergency Department
GA	Gestational Age
ICD	International Classification of Diseases
Min	Minimum
Max	Maximum
NACRS	Ontario Health National Ambulatory Care Reporting System
NICU	Neonatal Intensive Care Unit
PN	Patient Navigator
REDCap	Research Electronic Data Capture
RUQ	Resource Use Questionnaire
RUQ-4	Resource Use Questionnaire First Assessment
RUQ-12	Resource Use Questionnaire Second Assessment
SC	Standard Care
SD	Standard Deviation
USD	United States Dollars
WHO	World Health Organization

## Appendix A. Methods

### Appendix A.1. Data Management

A rigorous data management process was applied to all resource use questionnaire (RUQ) data to ensure accuracy, consistency, and completeness. To maintain temporal consistency in cost estimation and align with protocol-defined observation windows, data falling outside the 4-month and 12-month post-discharge assessment points were excluded based on prespecified decision rules, informed by the observed mean duration between assessment time points. For the first assessment (RUQ-4), a tolerance window of +4 weeks post-discharge was applied. Data collected beyond this window were either reassigned or excluded. For example, if RUQ-12 was completed at 5 months post-discharge and RUQ-4 was missing, RUQ-12 was reclassified as RUQ-4. For the second assessment, data were included only if collected within 10 months of the first assessment; otherwise, they were excluded. This approach ensured comparability across participants and minimized bias from differential follow-up durations. Before any exclusion, key demographic and infant medical history variables were compared and no statistical significance was found between excluded and included cases.

RUQ data were entered into Microsoft Excel, where validation checks were implemented to ensure consistency with predefined response categories and logical rules. Outliers (e.g., unusually high or low values in resource use) were flagged and reviewed in consultation with the research team. Datasets were securely stored. All data transformations and changes were documented in a working log file, and cleaned datasets were prepared for statistical analysis in accordance with a standardized set of decision rules.

### Appendix A.2. Costing

Resource use costs were categorized into nine domains: primary care provider visits, medical sub-specialist visits, allied health professional services, parental mental health service use, emergency department visits, infant’s medication, purchased equipment and materials and care coordination services. Detailed costing methods are provided in the Methods Supplement and Supplemental Material Figure S2.

To maintain uniform prices for costing, unit prices were collected from publicly available sources in Ontario, Canada, which had the highest study enrollment. Physician fees were obtained from the Ontario Schedule of Benefits [33] and medication unit prices were obtained from the Ontario Drug Formulary [34]. ED visit costs were collected using the IntelliHealth repository, administered by Ontario Ministry of Health [35]. The majority of prices were obtained for 2023 and adjusted to 2025 values using the Consumer Price Index for the Health and Personal Care sector obtained from Statistics Canada. While productivity time losses were captured, these were not monetized as they do not typically represent a cost to the public sector or to families, especially when parents are compensated with parental leave benefits in the first year after birth. A detailed costing table indicating unit prices for each service and resource is available in Supplemental Material Table S12.

### Appendix A.3. Sensitivity Analysis

A series of one-way deterministic sensitivity analyses were conducted to evaluate the robustness of the cost estimates and to explore the influence of key cost assumptions on the overall findings. These analyses focused on unit price variations and structural uncertainties related to intervention-specific components.

Seven one-way sensitivity analyses were conducted to assess the impact of uncertainty in key cost parameters. First, unit price rates for allied health professionals were varied within plausible ranges sourced from public fee schedules and published online estimates. This included services such as craniosacral therapy, chiropractic care, home nursing, and nursing care not provided under publicly funded home care programs. Second, parental mental health service costs were varied independently to account for differences in provider types and associated billing rates, including psychologists, social workers, mental health therapists or consultants, and marriage and family therapists. Third, wage assumptions for care coordination staff were adjusted to reflect differences in qualifications and employment models across sources, including Local Health Integration Network social workers, case managers, and staff associated with publicly funded programs such as Grandview Children’s Centre and Healthy Babies Healthy Children. Fourth, due to reported difficulties among parents in distinguishing between roles within the care team, specifically nurse navigators with patient navigators and care coordinators, a combined costing scenario was conducted. This analysis grouped these services under a unified category to assess the potential impact of role ambiguity on cost attribution and overall estimates.

For the intervention group, three additional one-way sensitivity analyses were performed specific to the nurse navigator. These included variation in the hourly wage, total time spent delivering services per family, and the allocation of the fixed startup cost by changing the assumed number of families served. Each analysis was conducted independently to isolate the impact of individual parameters on total and category-specific cost estimates.
