# Supplementary material for: Resource Use and Costs of Nurse Navigator Support for Parents of High-Risk Infants After Discharge from a Neonatal Intensive Care Unit
Source: Children (Basel). 2026 May 9;13(5):665. doi: 10.3390/children13050665 (PMC13204878; doi:10.3390/children13050665)
Supplement: Supplementary file 1 [file children-13-00665-s001.zip › Supplemental Figure S1_wu1.pdf]

Supplemental Figure S1. CCENT Trial Consort

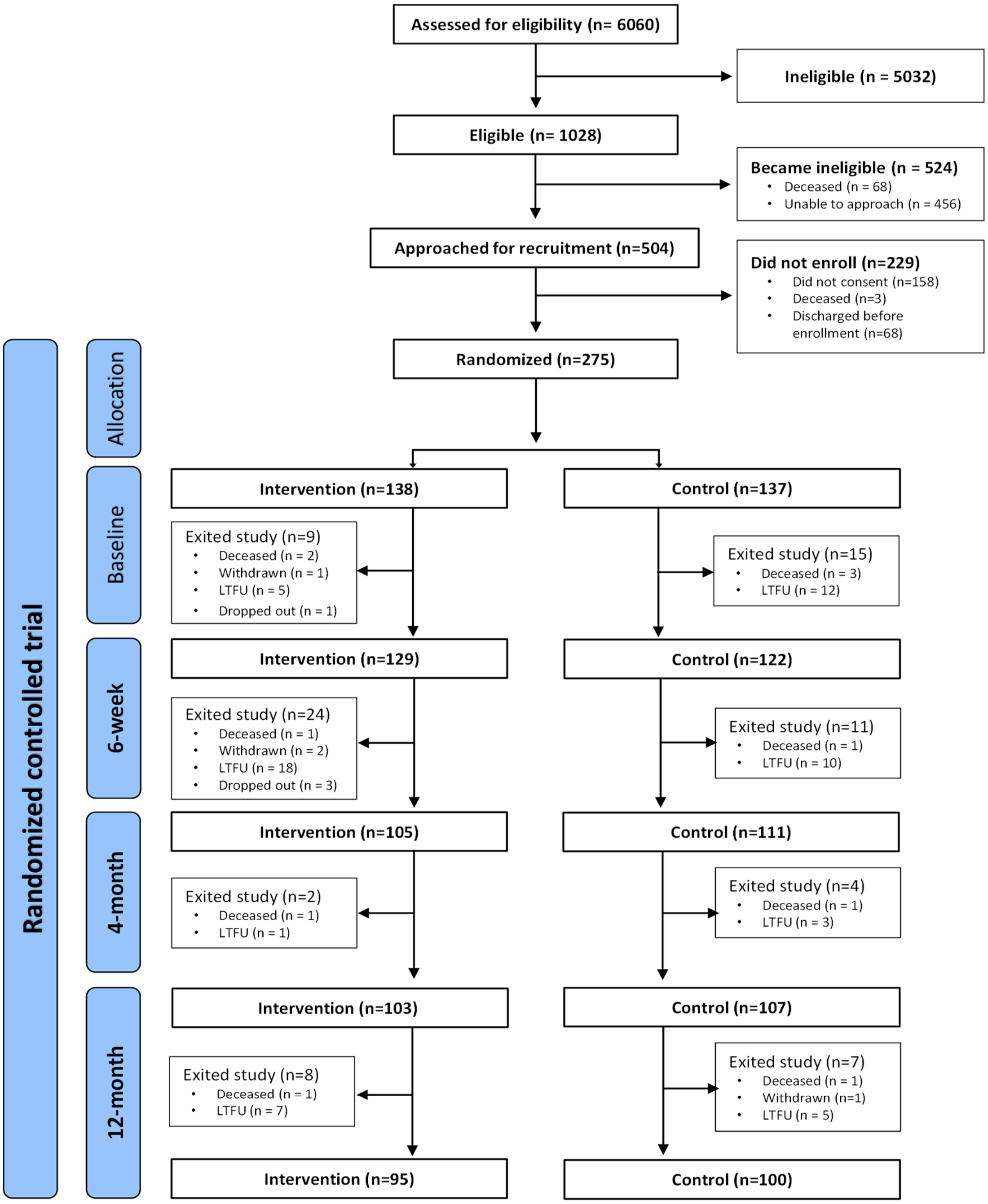

Abbreviations and definitions:
Lost to follow up (LTFU): Unable to contact participant after 3 calls and 3 e-mails
Dropped out: Parent has expressed that they would like to be removed from study
Withdrawn: Removed by study team due to ineligibility criteria post-enrollment

Resource Use and Cost

	Intervention	Control
Assessment 1 only	18	23
Assessment 2 only	22	22
Both Assessment 1 and 2	57	60
Total A1 completed	75	83
Total A2 completed	79	82
