# Supplementary material for: Resource Use and Costs of Nurse Navigator Support for Parents of High-Risk Infants After Discharge from a Neonatal Intensive Care Unit
Source: Children (Basel). 2026 May 9;13(5):665. doi: 10.3390/children13050665 (PMC13204878; doi:10.3390/children13050665)
Supplement: Supplementary file 1 [file children-13-00665-s001.zip › Supplementary Costing Methods_wu3.pdf]

Resource Use and Costs of Nurse Navigator Support for Caregivers of High-Risk Infants after Discharge from a Neonatal Intensive Care Unit

Supplementary Methods

Costing

Resource use costs were categorized into nine domains: primary care provider visits, medical subspecialist visits, allied health professional services, parental mental health service use, emergency department visits, infant's medication, purchased equipment and materials and care coordination services. A detailed costing table indicating unit prices for each service and resource is available in Supplemental Material Table 12.

For services provided by primary care providers and medical subspecialists, unit costs were obtained from the 2023 Ontario Schedule of Benefits [1]. A "general assessment" fee code was applied to initial visits, while a "general re-assessment" code was used for subsequent visits. If a service was delivered virtually, a fee code for "repeated consultation by video" was used. For medical subspecialist visits, an initial visit was assigned a "medical specific assessment" fee code. Follow-up visits were costed using the "medical specific re-assessment," "minor assessment," or "partial assessment" codes depending on the clinical context. A general consultation fee code was only used when none of the above codes were applicable. This approach was taken because consultation fees are typically higher and could lead to an overestimation of costs if used broadly. As a result, consultation codes were restricted to surgical specialties, including neurosurgery, plastic surgery, and general surgery. In cases where patients underwent DNA sequencing and received genetic consultation, a "clinical interpretation by a geneticist requested" fee code was applied at the corresponding fee-for-service rate. Total service costs were calculated for each patient by multiplying the frequency of each service by its corresponding fee code and summing across all service categories.

Similar to medical visits, infant allied health professional services and parental mental health service use were calculated by using reported frequency and corresponding rate. However, unit prices for these services were obtained from various sources, including public sector fee schedules, private provider listings, and online databases. In general, "initial" or "first visit" fees were applied to single visits, while "repeated" or "follow-up" visit fees were used for multiple visits. Given the variation in pricing by region and provider type, a multi-way sensitivity analysis was conducted to account for uncertainty in unit cost estimates.

Emergency department (ED) visits were also included in the cost analysis. For each reported ED visit, the parent's stated reason was mapped to a corresponding International Classification of Diseases, tenth revision (ICD-10) diagnosis code [2] based on the World Health Organization (WHO) classification [2]. Using the three-character main diagnosis ICD-10 code, visit-level costs were extracted from the Ontario Health National Ambulatory Care Reporting System (NACRS) database (accessed through IntelliHealth) [3] for the study period spanning 2018 to 2022. In cases where the reported reason was ambiguous (e.g., "bump head") and a direct match was unavailable in IntelliHealth, the broader upper branch two-character ICD-10 category was used to assign a cost estimate. For each ED visit cost, an ED physician fee was assigned using fee code "Multiple systems assessment."

Outpatient medication used by infants were costed based on several key factors, including quantity of use, patient weight, and drug price. Due to inconsistencies and missing information in parent-reported medication data, as well as the absence of direct weight measurements at the time of assessment, direct cost calculation based solely on reported data was deemed unreliable. To ensure consistency and accuracy, cost estimation incorporated standardized prescribing guidelines from the SickKids Pediatric Drug Formulary, weight projections based on the World Health Organization (WHO) Child Growth Standards [4], and drug prices obtained from the Ontario Drug Benefit Plan [5]. Medication dosage and regimen were determined using the SickKids Formulary, applying the lowest recommended dose for estimation. The reported duration of medication use was used; however, if the reported duration exceeded the interval between discharge and RUQ-4m or between RUQ-4m and RUQ-12m, it was capped at the maximum recommended duration for infants under 2 years of age as specified by the SickKids guidelines. As pediatric medication dosing is typically weight-based, and infant's weight was not directly collected in the RUQ-4m and RUQ-12m assessments and was not available from sources, estimated weight was derived using birth weight and age-adjusted growth models from the WHO Child Growth Standards [4]. For infants aged 0 to 24 months, weight was estimated using the formula: $W(t)=W(birth)\times(t/30)^p$, where t is age in days (i.e., age at each assessment time point) and p is a sex-specific growth parameter. According to the WHO, the growth parameter p varies by age and sex: for infants aged 0-6 months, p=0.285 for boys and p=0.276 for girls; for those aged 6 to 24 months, p=0.150 for boys and p=0.134 for girls. This weight estimation approach was applied to RUQ-4m calculations for both study groups. However, it was deemed inappropriate for RUQ-12m due to the unique characteristics of the study population. Specifically, some infants in the cohort were born with extremely low birth weight (<1 kg), and in several cases, the WHO growth model yielded implausible weight estimates at 12 months. For example, a male infant with a birth weight of 562 g was projected to weigh only 0.818 kg at one year—an estimate that is clinically unrealistic. To address this, estimated weight for RUQ-12 was obtained directly from the WHO growth percentile charts using the 50th percentile (median) weight for the child's age and sex.

A dispensing fee was applied to all medication use, and a compounding fee was applied when applicable.

Equipment and materials purchased to support the infant's health and well-being were reported by caregivers in the RUQ-4 and RUQ-12 questionnaires. To estimate associated costs, the total expenditure was calculated by summing the reported costs of all eligible items purchased within the observation periods. Only items deemed to have a direct or supportive role in improving the infant's health status were included in the analysis (e.g., specialized feeding equipment or respiratory support devices). Conversely, routine or non-health-enhancing items essential for basic infant care, such as car seats, strollers, general-purpose clothing, and diapers were excluded, as these are considered standard caregiving supplies rather than targeted health interventions.

Use of care coordination services aimed at helping families access healthcare, coordinate multidisciplinary care, and navigate the healthcare system (in addition to CCENT) were collected. Costs were calculated based on reported interactions with various professionals involved in the process, including social workers, care coordinators, case managers, patient navigators, and relevant programs. The cost for each care coordination service was determined by multiplying the reported time spent on each service by the corresponding hourly wage for each professional and

then summing the costs across all professionals to derive the total cost for each child. Care coordinator and patient navigator services were excluded from the reference case analysis for both the intervention and control groups due to ambiguous data entry, as parents might not have distinguished these services from those provided within CCENT.

References

1. Ontario Ministry of Health. Schedule of Benefits for Physician Services under the Health Insurance Act (June 29, 2023; effective July 24, 2023). Available online: https://www.ontario.ca/page/ohip-schedule-benefits-and-fees (accessed on 25 August 2023).
2. World Health Organization. International Statistical Classification of Diseases and Related Health Problems, 10th Revision (ICD-10) browser. Available online: https://icd.who.int/browse10/2019/en (accessed on 28 January 2026).
3. Ontario Ministry of Health. IntelliHealth Ontario. Available online: https://intellihealth.moh.gov.on.ca/ (accessed on 12 December 2023).
4. World Health Organization. Child Growth Standards. Available online: https://www.who.int/tools/child-growth-standards (accessed on 8 March 2024).
5. Ontario Ministry of Health. Ontario Drug Benefit Formulary and Comparative Drug Index. Available online: https://www.formulary.health.gov.on.ca/formulary/ (accessed on 13 May 2023).
